# Supplementary material for: Transcription regulates the spatio-temporal dynamics of genes through micro-compartmentalization
Source: Nat Commun. 2024 Jun 25;15:5393. doi: 10.1038/s41467-024-49727-7 (PMC11199603; doi:10.1038/s41467-024-49727-7)
Supplement: Supplementary file 3 — Description of Additional Supplementary Files [file 41467_2024_49727_MOESM3_ESM.pdf]

Description of Additional Supplementary Files

File Name: Supplementary Movie 1
Description: Movie showing a typical simulated trajectory of a 100-kb long gene (white monomers) along with two 100-kb long flanking regions (dark blue monomers). The red monomers represent RNA Pol IIbound loci within the genes. The average Pol II density is 0.5, E=-3kT and no restriction on interaction valency. The left panel shows the 3D organization of all the monomers while the right panel only shows the arrangement of Pol II-bound monomers. The lower panel provides a 1D representation of the location of Pol IIbound monomers within the gene. The movie depicts the condensation of Pol II-bound monomers, which subsequently facilitates the compaction of the full gene.

File Name: Supplementary Movie 2
Description: As in Supplementary Movie 1 but for a valency number 2. The movie illustrates how a smaller valency number destabilizes Pol II-mediated condensation, leading to a reduction in intragene compaction.

File Name: Supplementary Movie 3
Description: Movie demonstrating how bursty gene activity impacts on the structural dynamics of the gene. In the left panel, a typical simulated trajectory for a 200-kb long gene (Cyan monomers) with flanking regions (dark blue monomers).Monomers of different colors represent Pol II-bound regions belonging to different transcriptional trains. The right panel visualizes the spatial arrangement of Pol IIbound monomers from different trains, providing insights into their interplay. The lower panel shows the 1D arrangement of Pol II-bound monomers within the gene, highlighting their positioning. This movie illustrates how gene conformation is influenced and shaped by the dynamic intra- and intertrain interactions.

File Name: Supplementary Movie 4
Description: Movie illustrating inter-gene interactions. In the left panel, a simulated trajectory showing the behavior of two distinct genes (white and light blue monomers) with their surrounding genomic regions (dark blue monomers). Pol II-bound loci for each gene are represented by red and orange monomers respectively. The left panel provides insight into the spatial arrangement of Pol II-bound monomers belonging to different genes. The lower panel shows the 1D representation of the location of Pol IIbound monomers within these genes. This movie illustrates how intra- and inter-gene interactions are driven by dynamic Pol IImediated interactions, ultimately leading to the formation of transcriptionally-active subcompartments.
